# Subtle executive deficits are associated with higher brain amyloid burden and lower cortical volume in subjective cognitive decline: the FACEHBI cohort

**DOI:** 10.1038/s41598-020-74704-7

**Published:** 2020-10-20

**Authors:** Alba Pérez-Cordón, Gemma Monté-Rubio, Angela Sanabria, Octavio Rodriguez-Gomez, Sergi Valero, Carla Abdelnour, Marta Marquié, Ana Espinosa, Gemma Ortega, Isabel Hernandez, Maitee Rosende-Roca, Liliana Vargas, Ana Mauleón, Silvia Gil, Juan Pablo Tartari, Francisco Lomeña, Francisco Campos, Assumpta Vivas, Marta Gomez-Chiari, Alba Benaque, Agustin Ruiz, Luis Tárraga, Mercè Boada, Montserrat Alegret, FACEHBI Group, N. Aguilera, M. Berthier, M. Buendia, S. Bullich, P. Cañabate, C. Cuevas, I. de Rojas, A. Gailhajenet, S. Diego, J. Giménez, R. Gismondi, M. Guitart, M. Ibarria, A. Lafuente, E. Martín, J. Martínez, M. Moreno, S. Moreno-Grau, L. Núñez, A. Orellana, A. Páez, A. Pancho, J. Pavía, E. Pelejà, V. Pérez-Grijalba, P. Pesini, S. Preckler, J. Romero, L. Montrreal, M. Sarasa, O. Sotolongo-Grau, M. A. Tejero, M. Torres

**Affiliations:** 1 Research Center and Memory Clinic, Fundació ACE, Institut Català de Neurociències Aplicades, Universitat Internacional de Catalunya, C/ Gran Via de Carles III, 85 bis, 08028 Barcelona, Spain; 2 Networking Research Center on Neurodegenerative Diseases (CIBERNED), Instituto de Salud Carlos III, Madrid, Spain; 3 Servei de Medicina Nuclear, Hospital Clínic I Provincial, Barcelona, Spain; 4 Departament de Diagnòstic per la Imatge, Clínica Corachan, Barcelona, Spain; 5 Cognitive Neurology and Aphasia Unit (UNCA), University of Malaga, Malaga, Spain; 6 Piramal Imaging GmbH, Berlin, Germany; 7 Grifols, Barcelona, Spain; 8 Araclon Biothech, Saragossa, Spain

**Keywords:** Cognitive ageing, Neurodegenerative diseases, Diagnostic markers

## Abstract

To determine whether lower performance on executive function tests in subjective cognitive decline (SCD) individuals are associated with higher levels of brain amyloid beta (Aβ) deposition and regional volumetric reduction in areas of interest for Alzheimer’s disease (AD). 195 individuals with SCD from the FACEHBI study were assessed with a neuropsychological battery that included the following nine executive function tests: Trail Making Test A and B (TMTA, TMTB), the Rule Shift Cards subtest of BADS, the Automatic Inhibition subtest of the Syndrom Kurz Test (AI-SKT), Digit Span Backwards and Similarities from WAIS-III, and the letter, semantic, and verb fluency tests. All subjects underwent an 18F-Florbetaben positron emission tomography (FBB-PET) scan to measure global standard uptake value ratio (SUVR), and a magnetic resonance imaging (MRI). A multiple regression analysis, adjusted for age, was carried out to explore the association between global SUVR and performance on executive tests. Then, on those tests significantly associated with amyloid burden, a voxel-based morphometry (VBM) analysis was carried out to explore their correlates with grey matter volume. Multiple regression analysis revealed a statistically significant association between Aβ deposition and performance on one of the executive tests (the AI-SKT). Moreover, VBM analysis showed worse AI-SKT scores were related to lower volume in bilateral hippocampus and left inferior frontal regions. In conclusion, in SCD individuals, worse automatic inhibition ability has been found related to higher cerebral Aβ deposition and lower volume in the hippocampus and frontal regions. Thus, our results may contribute to the early detection of AD in individuals with SCD.

## Introduction

Subjective cognitive decline (SCD) refers to the self-perception of worsening of memory or other cognitive functions without objective impairment on existing standardized cognitive tests^1,2^. Cross-sectional studies in normal aging support SCD’s association with Alzheimer’s disease–related (AD) biomarker abnormalities such as amyloid burden^3,4^, reduced hippocampal volume^5,6^ and regional brain hypometabolism^7,8^. Moreover, longitudinal studies have demonstrated that individuals with SCD have an increased annual risk of conversion to mild cognitive impairment (MCI) and dementia^2,9^.

Several studies have found lower performance on episodic memory tests related to higher brain amyloid burden in cognitively healthy older adults^10–12^. However, memory does not seem the only cognitive function suffering an early decline in preclinical AD. Executive function performance can predict conversion to cognitive impairment^13–16^ and become an early sign of AD^17,18^.

Neuroimaging studies in healthy elderly have reported worse performance on executive tests associated with lower grey matter (GM) volume in frontal areas^19^. Lower volume in neocortical regions, such as the orbitofrontal, posterior cingulate and precuneus, correlates with higher amyloid burden in SCD, but not in healthy controls or in patients with MCI or AD dementia^20^. A recent study demonstrated that those individuals from a population-based cohort who presented more self-reporting cognitive decline had lower GM volume in known AD-related areas^21^.

The work presented herein is part of the *Fundació ACE Healthy Brain Initiative* (FACEHBI)^22^, a broad study based on a cohort of 200 middle-aged adults with SCD and focused on increasing the understanding of AD’s preclinical stages. It is a longitudinal study involving a multimodal biomarker approach intended to capture the more relevant molecular, structural and functional processes present in the earliest phases of AD combined with a comprehensive and sensitive neuropsychological protocol.

A better understanding of the relationship between AD biomarkers and objective cognitive performance in SCD is an active line of research. A previous study from our group using data from the FACEHBI cohort^12^ found that worse performance on the Face-Name Associative Memory test was related to higher amyloid beta (Aβ) deposition in individuals with SCD. Similarly, Amariglio et al.^3^ reported a significant association between higher Aβ deposition and worse performance on episodic and working memory tests. Since previous studies in cognitively healthy individuals have demonstrated that executive deficits can precede other cognitive ones, such as memory^18,23^, we decided to focus on executive functioning.

The concept of executive function includes a series of cognitive processes, such as impulse control, response inhibition, working memory, cognitive flexibility, planning, judgment and decision-making, which facilitate achieving a specific goal^24,25^. This current work focuses on determining whether or not a relationship exists between performance in a series of executive tests and AD biomarkers. In the present study, we hypothesized that executive function tests may be a useful tool to detect subtle cognitive deficits related to AD biomarkers in SCD individuals. The aims of this study were (1) to determine whether lower performance on executive function tests in subjective cognitive decline (SCD) individuals are associated with higher levels of brain Aβ deposition; and (2) in those executive function tests correlated with amyloid burden, to elucidate if worse performance is associated with a specific GM topology in regions known to be sensitive to AD.

## Results

### Sociodemographic and clinical data

The sociodemographic and clinical characteristics of 195 study participants with SCD are detailed in Table 1.

**Table 1:** General characteristics of the participants.

	FACEHBI group	Min–max
Sex (female n/%)	121 (62.00)	–
Age, in years (mean/SD)	65.71 (7.32)	51–86
Education, in years (mean/SD)	14.94 (4.65)	6–28
Vocabulary WAIS-III IQ (mean/SD)	43.95 (7.41)	27–61
MMSE (mean/SD)	29.21 (0.94)	27–30
Trail Making Test A (mean/SD)	43.86 (12.29)	18–141
Trail Making Test B (mean/SD)	100.10 (58.40)	34–394
BADS Rule Shift Card subtest (mean/SD)	2.99 (0.79)	0–4
Digit Span Backwards WAIS-III (mean/SD)	4.32 (0.95)	3–8
AI-SKT (mean/SD)	20.24 (4.18)	12–35
Letter verbal fluency (mean/SD)	18.00 (4.52)	9–39
Semantic verbal fluency (mean/SD)	21.29 (4.36)	12–36
Verb verbal fluency (mean/SD)	20.67 (6.10)	9–33
Similarities WAIS-III (mean/SD)	13.90 (1.24)	10–15
Global SUVR (mean/SD)	1.22 (0.14)	0.99–1.98
APOEε4 (n/%)	50 (25.60)	–
FBB-PET + (n/%)	16 (8.00)	–

*Max* maximum, *Min* minimum, *SD* standard deviation, *MMSE* Mini-mental State Examination, *BADS* Behavioral Assessment of the Dysexecutive Syndrome, *AI-SKT* Automatic Inhibition subtest of the Syndrom Kurz Test, *Vocabulary WAIS-III IQ* Vocabulary subtest from the Wechsler Adult Intelligence Scale, Third Edition, *FBB-PET* 18F-Florbetaben positron emission tomography. FBB-PET+ indicates amyloid-positivity. A cut-off SUVR value of 1.45 was selected as amyloid positive criterion, that is, to classify subjects in FBB-PET positive and FBB-PET negative groups.

### Correlations between Aβ deposition and executive function performance

**Figure 1:**
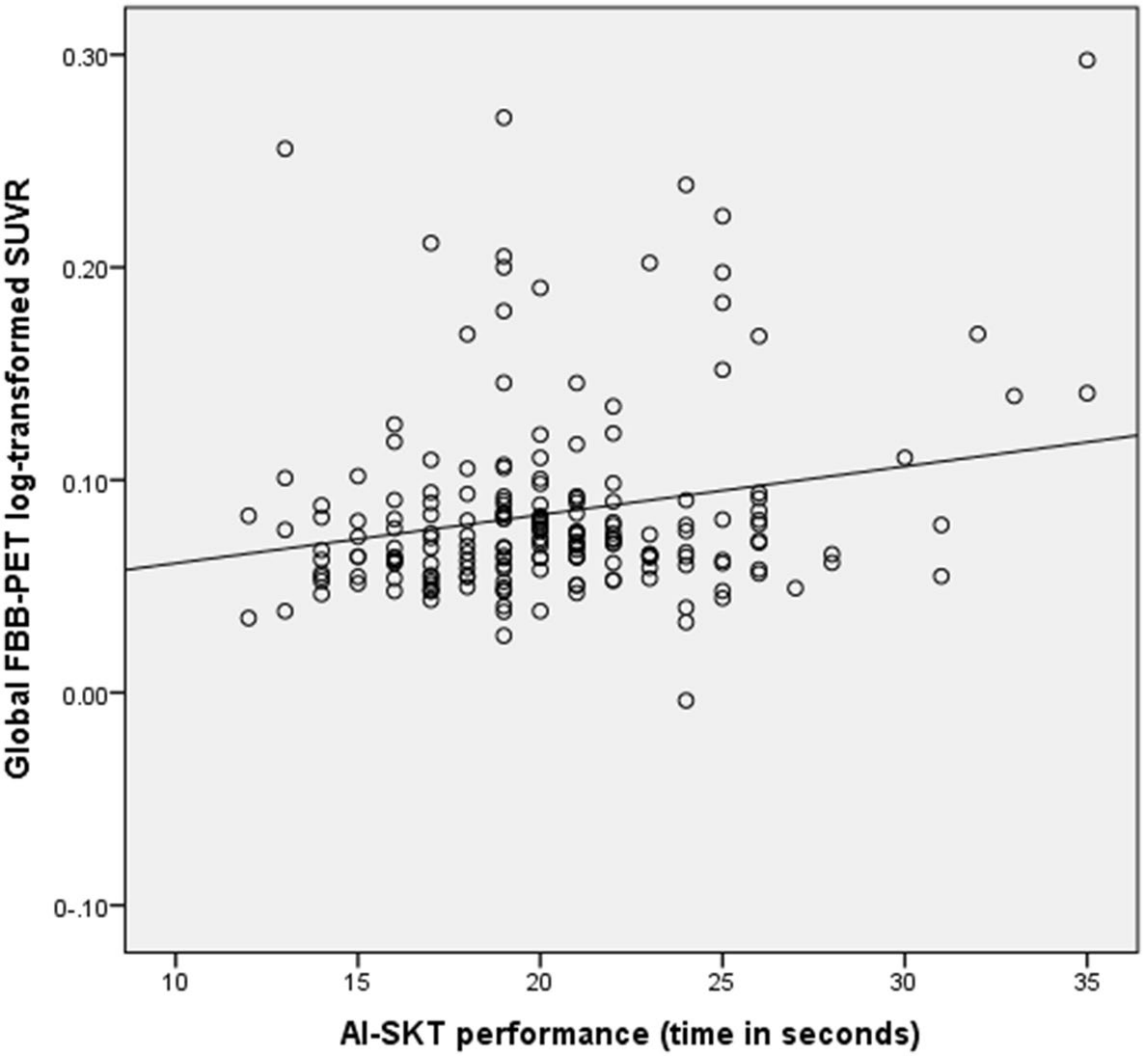
Plotting Global FBB-PET SUVR against AI-SKT performance.

Multiple regression analysis was performed using as an outcome the global SUVR. Predictors of the model were all executive function variables of the study: Rule Shift Cards from BADS, Digit Span Backwards and Similarities from WAIS-III, Trail Making Test A and B, Automatic Inhibition subtest of the Syndrome Kurz Test (AI-SKT), and letter, semantic and verb fluency tests. Age was included in the model as an adjusting variable because it is the only demographic variable (age, sex, years of formal education) that obtained a statistically significant association with global SUVR (r = 0.21, *p* = 0.003). Under a stepwise procedure, only the AI-SKT remained as a statistically significant variable of the model (β = 0.163, *p* = 0.028) (see Table 2; Fig. 1).

**Table 2:** Multiple regression analyses of AI-SKT scores with FBB-PET SUVR in Global cortex, adjusted by age.

	Global SUVR		
	b	β	*p*
Constant	− 0.016		0.596
Age (years)	0.001	0.158	0.034*
AI-SKT (time in seconds)	0.002	0.163	0.028*

*AI-SKT* Automatic Inhibition from the Syndrom Kurz Test, *SUVR* Standard uptake value ratio.

*p < 0.05.

### Correlations between GM volume and AI-SKT performance: VBM analyses

**Figure 2:**
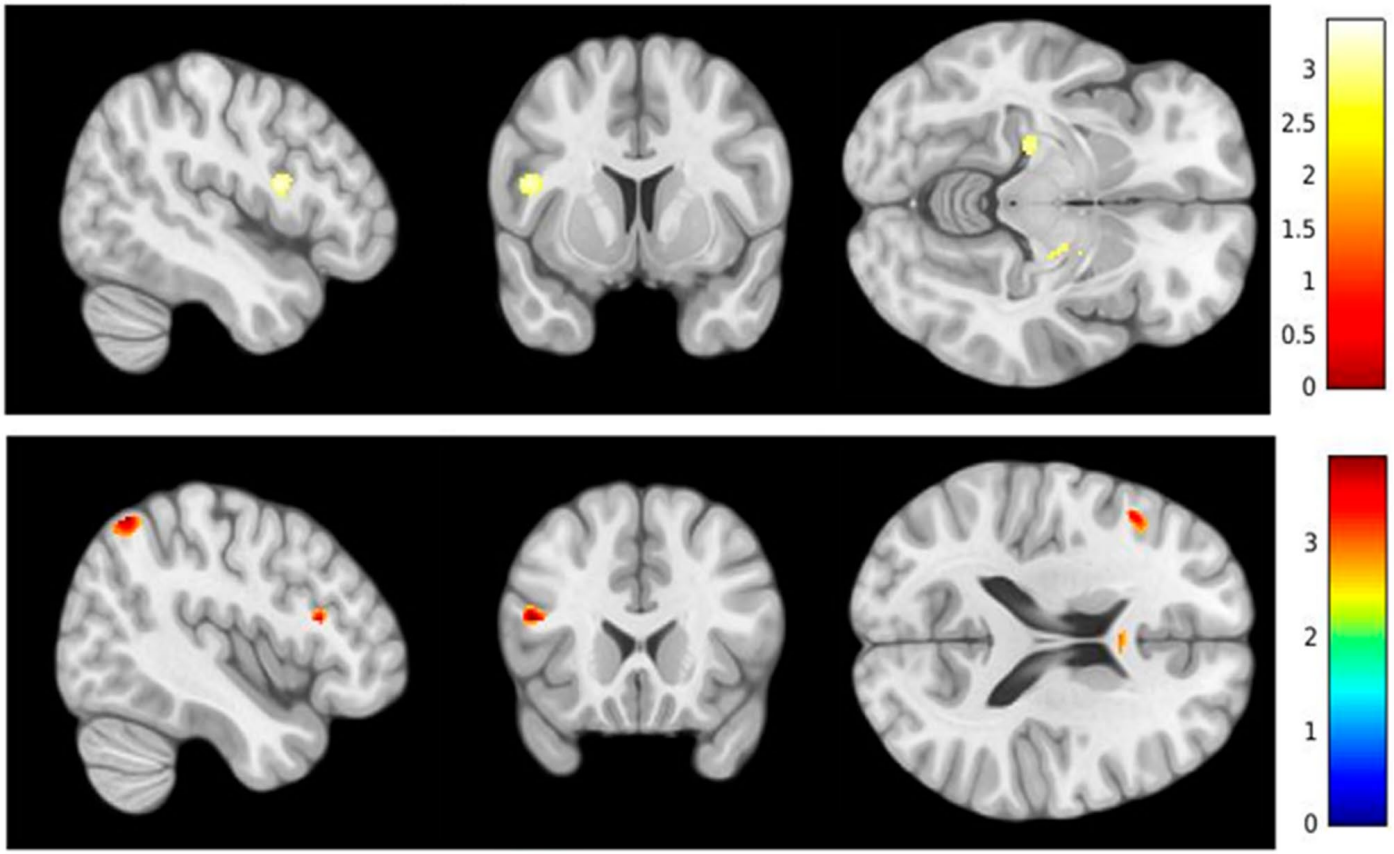
Lower (top) and higher (bottom) GM volume found in association with worse performance on the AI-SKT subtest.

As the AI-SKT was the only executive test with a statistically significant association with Aβ deposition in the former analysis, it was the only test selected for VBM. Higher GM volume in the left frontal inferior, parietal, angular, and anterior cingulate was found related to worse AI-SKT scores (Table 3). Moreover, lower GM volume in the bilateral hippocampus and the left frontal, Rolandic, and precentral regions was found associated with worse AI-SKT performance. Anatomical regions and statistics from both contrasts are detailed in Table 3 (Fig. 2).

**Table 3:** Locations and statistical details of GM volume changes in association with AI-SKT.

	MNI locations		k	Peak MNI coordinate			T-score
				X	Y	z	
AI-SKT positive association	Cluster 1	Parietal Inferior L	152	− 43	− 61	55	3.82
		Angular L					
	Cluster 2	Corpus Callosum	135	0	9	22	3.19
		Cingulum Anterior L					
	Cluster 3	Frontal Inferior L	104	− 48	19	18	3.9
AI-SKT negative association	Cluster 1	Hippocampus R	253	13	− 10	− 13	3.45
	Cluster 3	Frontal Inferior Operculum L	136	− 46	6	16	3.3
		Precental L					
		Rolandic Operculum L					
	Cluster 2	Hippocampus L	131	− 24	− 25	− 7	3.25

Negative association involved lower GM volume. Instead of a p-value, the T-score is provided for each cluster. It measures the size of the difference relative to data variation; this means that T is the calculated difference in units of standard error. As the magnitude of T increases, the evidence against the null hypothesis increases.

*AI-SKT* Automatic Inhibition from the Syndrom Kurz Test, *R* right, *L* left.

## Discussion

The results of the present study reinforce the importance of assessing executive functioning in the early detection of AD in individuals with SCD. We found lower performance on the AI-SKT executive subtest to be related to higher brain Aβ deposition. For the rest of the executive function tests no association between brain Aβ deposition and performance was found. This finding may be explained by the fact that these tests assess different cognitive processes related to executive functions such as working memory, cognitive flexibility or inhibition ability^24,25^. Additionally, low AI-SKT scores were associated with higher GM volume in the left frontal inferior, parietal, angular, and anterior cingulate areas and lower GM volume in the bilateral hippocampus and left frontal regions.

In individuals with SCD, we found poorer automatic inhibition ability related to higher brain Aβ deposition. Consistent with this result, Snitz et al.^18^ reported that Aβ-positive older adults without dementia showed evidence of a greater decline in executive function over an interval of 7–9 years prior to neuroimaging, compared with Aβ-negative individuals. Moreover, in a cross-sectional study using CSF as a marker, Harrington et al.^23^ found subtle executive dysfunction as one of the first deficits in preclinical AD.

With regard to the structural MRI analysis, our data showed that worse AI-SKT subtest scores were related to lower volume of the bilateral hippocampus and left frontal regions (including the frontal inferior operculum, rolandic operculum, and precentral areas). It is well-known that the hippocampus is one of the earliest brain regions affected in AD according to the topographic progression of tau deposits and neurodegeneration^26,27^. In this sense, a decreased hippocampal volume is a well-established neuroimaging biomarker linked to the initial stages of AD^28,29^ and described as a predictor of conversion from MCI to dementia^30^. Our findings are consistent with those of other studies that have reported hippocampal volume changes in SCD^6,31,32^. Also in line with our findings , an association between a lower volume of hippocampus and the performance of executive functions has been described^33^. With regard to the association between worse AI-SKT subtest performance and lower GM volume in the left frontal regions, our results are consistent with those of previous studies in which set-shifting task performance was found to be related to the activation of the frontal inferior and precentral regions^34^.

Interestingly, we found a negative correlation between worse AI-SKT performance and higher volume in brain association areas (angular gyrus, cingulum anterior, corpus callosum) and the inferior frontal and parietal regions. Chételat et al.^35^ reported increased temporal lobe volume in healthy individuals who had high amyloid burden compared to those with low Aβ deposition. It has been argued that this may reflect a compensatory response to the local amyloid deposition. Another interpretation for the current findings might be related to the existence of a non-linear pattern of GM volume changes associated with early stages of AD. Gispert et al.^36^ analyzed the association between GM volume and AD CSF biomarkers and identified a nonlinear relationship between GM volume reduction and the progression of AD pathology.

We acknowledge that the present study has strengths and limitations. First, it is a cross-sectional study, so the results only reflect data from a particular point in time (the baseline visit of the study). As FACEHBI includes a follow-up with repeated cognitive testing, PET-FBB, and brain MRI every two years, we plan to continue our research about the longitudinal relationship between executive functions and AD biomarkers. The FACEHBI is a single-center study, that is, participants were exclusively recruited from Fundació ACE’s memory clinic, with all cognitive and biomarkers measures performed, collected, and processed in a homogeneous way, which guarantees data uniformity. This study was focused on determining whether a relationship between performance on executive tests and AD biomarkers could be detected, in analogy with previous literature on the relationship between memory tests and AD biomarkers. Therefore, the relationships of amyloid and GM volume with cognitive areas other than executive functions have not been explored in the work presented here.

The ultimate aim of the present study was to identify which executive function test may prove to be a sensitive tool to detect early markers of AD such as elevated amyloid burden or GM volume changes. However, the results of the present study might be interpreted with caution because it was performed in individuals with SCD with relatively preserved executive performance and low Aβ deposition in the brain. It will be important to check how these executive tests perform in different memory clinics and in community-based studies. Further studies are needed to find economical and noninvasive tools that could enable AD risk prediction in cognitively healthy individuals. To achieve this, we consider it crucial to perform additional longitudinal analyses to deepen our knowledge of preclinical AD.

In conclusion, in SCD individuals, a decline in automatic inhibition ability has been found to be associated with higher cerebral Aβ levels and lower volume in the hippocampus and frontal regions. These findings reinforce the importance of assessing executive functioning in individuals with SCD.

## Methods

### Subjects

From a cohort of 200 SCD subjects, 5 were excluded due to acquisition or movement artifacts in MRI or PET data. The resulting sample of 195 individuals had completed data from executive tests and was included in the study. The FACEHBI inclusion criteria are described in detail elsewhere^22^. In brief, they include: age older than 49 years old, literate with at least elementary school (or at least six years of formal education), Subjective Cognitive Complaints defined as a score of ≥ 8 on the Spanish Modified Questionnaire of Memory Failures in Everyday (MFE-30)^37^; a preserved performance on the Mini-mental State Examination (MMSE ≥ 27)^38,39^; a strictly normal performance for age and education in a comprehensive neuropsychological battery of Fundació ACE (NBACE)^40^; a Clinical Dementia Rating Score (CDR) of 0^41^; and without relevant depressive/anxiety symptoms on the Spanish version of the Hospital Anxiety and Depression Scale (HADS < 11)^42^, or another psychiatric illness. Exclusion criteria were: evidence of impairment in daily life activities, history of alcoholism and epilepsy; renal or liver failure; and presence of severe auditory or visual abnormalities that could affect neuropsychological test performance (see Rodriguez-Gomez et al., for details)^22^.

All patients underwent a complete neurological and neuropsychological examination, a set of self-administered questionnaires and a battery of multimodal biomarkers, including APOE genotyping, an MRI protocol and an ^18^F-Florbetaben PET (FBB-PET)^22^. Invalid FBB-PET and MRI data (i.e., acquisition or movement artifacts) were excluded from the current study.

### Neuropsychological assessment

All subjects underwent a comprehensive neuropsychological evaluation which included the NBACE^40^ and additional neuropsychological tests. For the purpose of the present study, only nine tests sensitive to executive functioning were analyzed: (1) Trail Making Test A (TMTA)^43^ consists of 25 circles numbered 1 to 25 spread over a sheet of paper. It requires drawing lines as quickly as possible connecting the numbers in ascending order. The time in seconds needed to complete the task was registered to measure visuomotor processing speed; (2) Trail Making Test B (TMTB)^43^ includes circles with both numbers (1–13) and letters (A–L). It requires drawing lines to connect the circles in an ascending order, but alternating between the numbers and letters. The time in seconds to finish the test was registered to assess sequencing ability; (3) Rule Shift Cards from the Behavioral Assessment of the Dysexecutive Syndrome (BADS)^44^ has two parts. Firstly, subjects were instructed to answer “YES” when shown a red card and “NO” when shown a black one; in the second part, they were instructed to say aloud “YES” if two sequential cards were the same color and “NO” if the colors were different to measure task switching abilities; (4) Verb verbal Fluency^45^, asked participants to name as many verbs in infinitive or actions as possible in one minute. They had to say the verbs in infinitive, but if they conjugated or repeated a verb, only the first one was considered as correct; (5) AI-SKT^46^ asks participants to read 2 lines with only the letters A and B and to say aloud “B” when reading an “A” and vice versa. The time of execution and errors were registered to assess the ability to inhibit automatic responses; (6) Letter Verbal Fluency^47^ asked participants to say aloud as many words as possible beginning with “P” in one minute; (7) Semantic Verbal Fluency^48^ asked participants to say aloud as many animals as possible in one minute; (8) Digit Span Backwards of the Wechsler Adult Intelligence Scale-Third Edition (WAIS-III)^49^ asked participants to repeat sequences of numbers in reverse order so as to measure working memory; (9) Similarities subtest of WAIS-III^49^ was presented with 10 word pairs and asked how they were alike to assess verbal reasoning.

### MRI and FBB-PET acquisitions

All MRI scans were acquired prior to FBB-PET. MRI was performed on a 1.5 T Siemens Magnetom Aera (Erlangen, Germany) using a 32-channel head coil. T1-weighted images were acquired using a rapid acquisition gradient-echo 3-D MPRAGE sequence with the following parameters: TR 2200 ms, TE 2.66 ms, TI 900 ms, slip angle 8º, FOV 250 mm, slice thickness 1 mm, and isotropic voxel size 1 × 1 × 1 mm. In addition, all participants underwent an axial T2-weighted, 3-D isotropic FLAIR and axial T2*-weighted sequences to detect significant vascular pathology or microbleeds.

FBB-PET images were acquired in a 90-day window after the baseline visit in a Siemens Biograph molecular-CT machine. Four FBB-PET scans of 5 min were acquired 90 min post IV injection of 300 Mbq of ^18^F-Florbetaben radio tracer (NeuraCeq), provided by Life Molecular Imaging (formerly Piramal Imaging).

### Standard uptake value ratio

First, MRI cortical and subcortical segmentation of the T1-weighted images was carried out with Freesurfer 5.3 (https://surfer.nmr.mgh.harvard.edu/). FBB-PET scans were processed with the FSL 5.0 package (https://fsl.fmrib.ox.ac.uk/fsl/fslwiki). The FBB-PET images were coregistered onto structural images, and then the standard uptake value ratio (SUVR) was determined as the mean value of the cortical regions segmented on MRI, and normalized by the cerebellum as the reference region.

### Voxel-based morphometry

A voxel-based morphometry (VBM)^50^ was conducted with the T1-weighted images. First, data were visually inspected for artefacts and manually centered in the anterior commissure (AC). Next, segmentation into grey matter (GM), white matter (WM) and other tissues was carried out using SPM12 (https://www.fil.ion.ucl.ac.uk/spm/software/spm12/). GM data were normalized to the MNI space according to the Dartel technique^51^, also implemented in SPM12. Finally, Jacobian-scaled GM images were smoothed through an isotropic Gaussian kernel using 6mm^3^ FWHM.

### Statistical analysis

#### Clinical variables in association with SUVR

Statistical analysis was performed using SPSS (version 26, SPSS Inc., Chicago, IL). All data were examined for normality, skew, and range restriction. SUVR was log10 transformed to normalize its distribution. Correlations or T-test were calculated for the demographic with global SUVR. In order to determine which executive functions (Rule Shift Cards from BADS, Digit Span Backwards and Similarities from WAIS-III, Trail Making Test A and B, Automatic Inhibition subtest of the Syndrome Kurz Test and letter, semantic, and verb verbal fluency tests) were statistically associated with global SUVR a multiple regression analysis was executed. Stepwise procedure was used to identify the final significant variables. Age was here included as an adjusting variable.

#### Topological GM correlates of executive test performances

Next, those executive function test scores which correlated to amyloid burden in the former step were explored separately in association with GM volume, using the VBM voxel-wise approach. The statistical associations between several variables (age, sex, SUVR, years of formal education and APOE status) and executive tests scores were assessed to include those variables with a significant association into the model. As the formula below shows, the final model design included age, sex and years of formal education as covariates. Statistical designs were also corrected for Total Intracranial Volume (TIV) as a global, and μ indicates the mean.

$$\upmu + {\text{sex}} + {\text{age}} + {\text{years}}\,{\text{of}}\,{\text{education}} + {\text{test}}\,{\text{performance}} + {\text{TIV}} $$

Statistical parametric maps were thresholded using uncorrected *p* < 0.005, and an extent threshold of k = 100 voxels per cluster.

### Ethical standards

All the participants signed written informed consent prior to any evaluation. The FACEHBI protocol received approval from the ethics committees of Hospital Clínic i Provincial in Barcelona, Spain (EudraCT number 2014-00079-38). The referral center ethics committee approved patient recruitment, and collection protocols were in accordance with ethical standards according to the World Medical Association Declaration of Helsinki—Ethical Principles for Medical Research Involving Human Subjects.

## Data Availability

Data used for this study are available from the corresponding author on reasonable request.
